# Gene self-control: when pre-mRNA splicing variants become competing endogenous RNAs

**DOI:** 10.3389/fgene.2014.00405

**Published:** 2014-11-19

**Authors:** Zhi-xiang Lu

**Affiliations:** Department of Microbiology, Immunology, and Molecular Genetics, University of California Los AngelesLos Angeles, CA, USA

**Keywords:** alternative splicing, nonsense-mediated decay, competing endogenous RNAs, non-coding RNAs, CDKAL1

Since Richard J. Roberts and Phillip A. Sharp discovered split genes (genes are interrupted by RNA-encoding regions called exons and non-coding segments called introns in eukaryotic genome) in 1970's, scientists have been finding many genes can generate more than one mRNA transcripts through AS (alternative splicing, e.g., by different exon-exon combination). This AS strategy increases protein repertoire, encodes proteins with diverse and sometimes even antagonistic activities (Kelemen et al., 2013). A new study led by Dr. Kazuhito Tomizawa and first author Bo Zhou from Kumamoto University in Japan reports that *CDKAL1-v1* (Cdk5 Regulator Subunit Associated Protein 1-Like), one splicing variant of *CDKAL1*, has no coding ability but acts as a miRNA sponge RNA, which regulates its full-length CDKAL1 protein (Zhou et al., 2014). Their results give us a unique paradigm of how AS possesses a regulatory role in controlling gene expression.

In addition to functioning as an “internal paralog” to deliver protein-coding message (Modrek and Lee, 2003), the main well-known mechanism of gene regulation by AS is alternative splicing-coupled nonsense-mediated decay (AS-NMD). Briefly, Pre-mRNA alternative splicing creates unstable mRNA isoforms with PTC (premature termination codon). Generally, if a PTC site is more than ~50 nucleotides upstream of the last exon-exon junction, this RNA isoform will be degraded by NMD, an RNA surveillance pathway to clean up splicing errors which may lead to damaging truncated proteins (Sibley, 2014). For example, PTBP1 (polypyrimidine tract-binding protein 1) is one typical splicing regulator; it can regulate its own gene level through *PTBP1*-dependent exon 11 skipping to generate an AS-NMD transcript (Wollerton et al., 2004). The auto regulation through this negative-feedback loop fine-tunes PTBP1 protein level in normal development (Figure 1A). Zhou et al.'s study reveals a new mechanism where AS can regulate its own gene via competition for common miRNAs. Like regulation of PTEN functional protein by crosstalk from both pseudogene *PTENP1*, and cognate genes by competing for common miRNAs of tumor suppressor PTEN (Poliseno et al., 2010; Tay et al., 2011), AS offers a simpler way to generate ceRNAs (competing endogenous RNAs) by indirectly regulating its own gene in this coding-independent manner. Zhou et al. find that although *CDKAL1-v1* is a short splicing variant which contains a PTC, it is not subjected to NMD. Interestingly, this non-coding RNA has the same targeting miRNA as full length *CDKAL1*, of which is a type 2 diabetes risk factor associated with insulin secretion. By competing miRNA bindings, *CDKAL1-v1* RNA level displays concordant expression pattern with the full length *CDKAL1* mRNA and protein levels (Figure 1B). Small interfering RNAs knockdown of *CDKAL1-v1* markedly reduces the full *CDKAL1* level or vice versa. Their data suggest *CDKAL1-v1*-mediated *CDKAL1* gene may underlie type 2 diabetes pathogenesis. Here I term such AS regulatory mechanism as alternative splicing-coupled competing endogenous RNAs (AS-ceRNAs) (Figure 1B).

**Figure 1:**
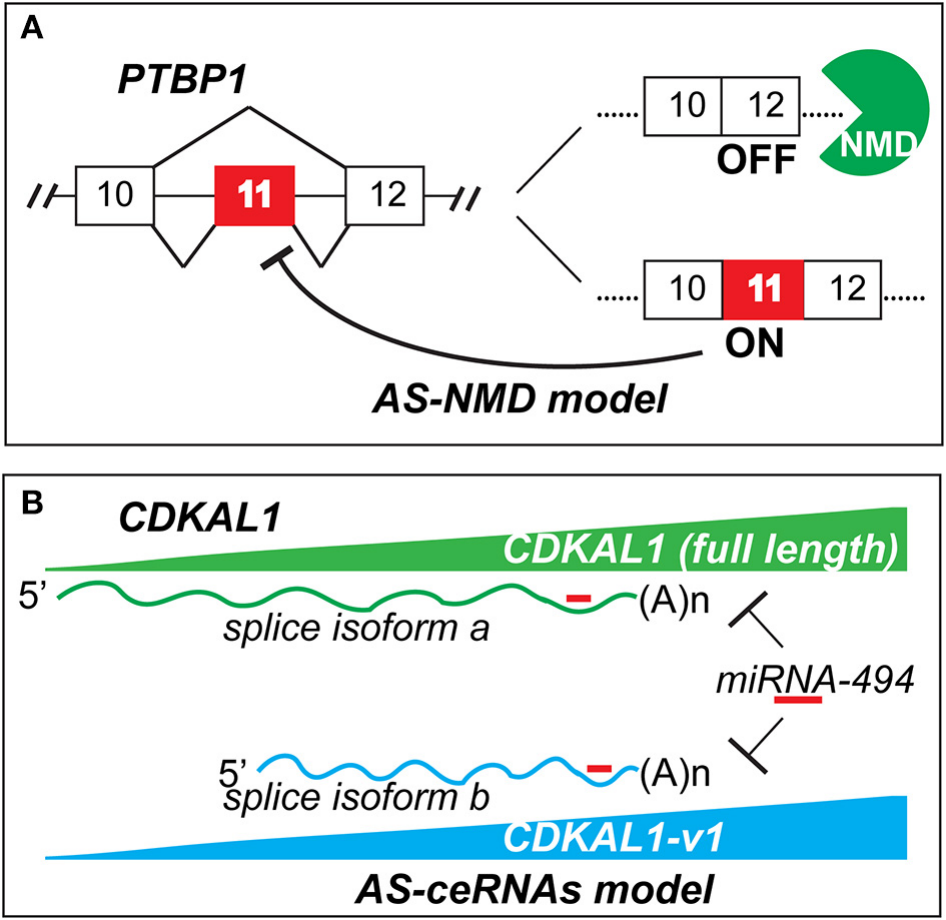
**AS-NMD (A) and AS-ceRNAs (B) models of gene self-control**. AS-NMD: alternative splicing-coupled nonsense-mediated decay; AS-ceRNAs: alternative splicing-coupled competing endogenous RNAs.

*CDKAL-v1* study by Zhou et al. (2014) opens an intriguing new possibility, and elucidates a critical but understudied layer of AS role in gene control. Recent estimate by next-generation RNA sequencing uncovers more than 90% multi-exon human genes undergoing alternative splicing (Pan et al., 2008; Wang et al., 2008), but to answer how many of splicing variants are functional, not results of “aberrant splicing” or “noisy splicing,” is still challenging. Post-transcriptional regulation by AS-ceRNAs model will encourage us to reevaluate the regulatory role of those uncharacterized splicing transcripts in reciprocal interactions with their own or cognate genes. RNA isoforms acting as ceRNAs such as *CDKAL1-v1* may become splicing correcting targets for therapeutic development.

## Conflict of interest statement

The author declares that the research was conducted in the absence of any commercial or financial relationships that could be construed as a potential conflict of interest.
